# DNA methylation age from peripheral blood predicts progression to Alzheimer’s disease, white matter disease burden, and cortical atrophy

**DOI:** 10.21203/rs.3.rs-5273529/v1

**Published:** 2024-10-28

**Authors:** Luke Bonham, Daniel Sirkis, Alina Pang, Leo Sugrue, Hernando Santamaría-García, Agustin Ibanez, Bruce Miller, Jennifer Yokoyama, Michael Corley

**Affiliations:** Department of Radiology and Biomedical Imaging, University of California, San Francisco; Memory and Aging Center, Department of Neurology, Weill Institute for Neurosciences, University of California, San Francisco; Division of Infectious Diseases, Department of Medicine, Weill Cornell Medicine; Department of Radiology and Biomedical Imaging, University of California, San Francisco; Pontificia Universidad Javeriana, Physiology and Psychiatry Departments; Universidad de San Andrés; Memory and Aging Center, Department of Neurology, Weill Institute for Neurosciences, University of California, San Francisco; Memory and Aging Center, Department of Neurology, Weill Institute for Neurosciences, University of California, San Francisco; Division of Infectious Diseases, Department of Medicine, Weill Cornell Medicine

**Keywords:** DNA methylation, epigenetic clock, neuroimaging

## Abstract

Cross-sectional studies suggest a limited relationship between accelerated epigenetic aging derived from epigenetic clocks, and Alzheimer’s disease (AD) pathophysiology or risk. However, most prior analyses have not utilized longitudinal analyses or whole-brain neuroimaging biomarkers of AD. Herein, we employed longitudinal modeling and structural neuroimaging analyses to test the hypothesis that accelerated epigenetic aging would predict AD progression. Using survival analyses, we found that two second generation epigenetic clocks, DNAmPhenoAge and DNAmGrimAge, predicted progression from cognitively normal aging to mild cognitive impairment or AD and worse longitudinal cognitive outcomes. Epigenetic age was also strongly associated with cortical thinning in AD-relevant regions and white matter disease burden. Thus, in contrast to earlier work suggesting limited applicability of blood-based epigenetic clocks in AD, our novel analytic framework suggests that second-generation epigenetic clocks have broad utility and may represent promising predictors of AD risk and pathophysiology.

## INTRODUCTION

Converging evidence from the study of model organisms and human brain tissue suggests that epigenetic aging – a metric of biologic age based on DNA methylation patterns – plays a critical role in Alzheimer’s disease (AD) pathophysiology. Briefly defined, epigenetics and epigenetic aging are measurable changes to DNA (often via DNA methylation) that occur over the lifespan which are regulated by genetic variation, behavior, environment, and human disease. Transgenic mouse models of AD demonstrate unique epigenetic alterations associated with AD pathology^1,2^ and studies of human brain tissue show marked DNA methylation differences in AD when compared to normal aging brain^3,4^. By contrast, data from clinical studies has been mixed. For example, a recent systematic review largely using cross-sectional studies found no strong evidence that epigenetic age estimates were associated with risk for dementia or mild cognitive impairment (MCI),^5^ while other smaller studies have suggested a limited though promising relationship with risk for AD or related disease biomarkers^4,6^.

There are multiple possible explanations for these surprisingly discrepant findings as insights from mouse models and postmortem human tissue are translated into clinical settings. First, most clinical studies performed to date have been cross-sectional rather than longitudinal – limiting evaluation for disease risk-modifying effects which may not appear on same-visit cognitive scores or biomarker measurements. Second, multiple generations of epigenetic clocks have been developed for analysis, with each generation and associated score based on distinct analytic underpinnings and resulting in distinct interpretations – making comparison and replication of results challenging. Finally, many studies have focused on one or two related outcomes such as disease status and cognitive scores, which may not be sensitive to interindividual differences in disease trajectory. Taken together, these findings suggest the need for additional studies using more sensitive longitudinal and biomarker data paired with well-established and validated epigenetic clocks.

Therefore, we investigated the relationship between two well-validated second-generation epigenetic clocks, DNAmPhenoAge and DNAmGrimAge^7–9^, and risk for MCI or AD using longitudinal analyses and multimodal neuroimaging. Specifically, we analyzed the rate of progression from cognitively normal (CN) aging to either MCI or AD, cortical thinning and white matter hyperintensities (WMH) on magnetic resonance imaging (MRI), and longitudinal cognitive changes. We hypothesized that combined longitudinal and multimodal imaging analyses would increase our ability to detect the modulation of AD risk by epigenetic age and that epigenetic age would predict AD risk independent of chronologic age.

## METHODS

### Participant Characteristics

This study utilized samples from 404 participants from the Alzheimer’s Disease Neuroimaging Initiative (ADNI) study with DNA methylation and structural neuroimaging data available. At baseline, 121 participants were considered CN, 236 were diagnosed with MCI, and 47 were diagnosed with AD. ADNI is a longitudinal study of subjects across the US that includes multimodal neuroimaging, blood biomarkers, and clinical markers of AD and has been described previously^10,11^. As part of the study, patients undergo a rigorous clinical exam, which includes neurologic examination, neuropsychiatric evaluation, cognitive testing, blood sampling, and brain MRI^12^. Participants were diagnosed as CN or with MCI or AD based on a structured protocol that integrated clinical data, cognitive testing, and brain MRI results, which has been described previously^13^. Clinical characteristics are detailed in Table 1. Clinical symptoms severity was measured using the Clinical Dementia Rating Scale Sum of Boxes (CDR-SB) score^14^. Cognitive changes over time were measured using the Montreal Cognitive Assessment (MoCA)^15^. Written and informed consent was obtained from study participants in compliance with local IRB and ADNI protocols as has been previously described^16^.

### Image Processing

Structural MRI images from all participants were segmented using two pipelines to evaluate cortical structure and white matter disease. Cortical segmentation and structural analysis was performed using FreeSurfer version 5.1 using T1-weighted images as previously described^17,18^. Briefly, all images were segmented using FreeSurfer’s automated pipeline and then manually checked for segmentation accuracy, with segmentation errors manually corrected^19^. Cortical thickness measurements from 68 cortical regions of interest (ROI; 34 for each hemisphere) in the Desikan-Killiany atlas^18^ were retained for analysis. White matter disease was estimated using a pipeline developed at the University of California, Davis, which quantifies WMH volumes via Bayesian modeling based on participants’ 3D T1 and FLAIR images^20^.

### DNA Methylation Assessment

DNA methylation was profiled from blood samples of ADNI participants using the Illumina Infinium Human MethylationEPIC V1 BeadChip Array, which covers ~ 866,000 CpGs (illumina.com). Briefly, data was normalized using the dasen method in the wateRmelon R package^21^, and quality control procedures were performed, including removal of samples with abnormal CpG detection p-values > 0.05, checking the ratio of X/Y chromosome probe intensities for sex concordance, and comparison of targeted SNP genotypes to genotype microarray data as previously described^22^. Estimates of epigenetic age for the Levine 513 CpG site DNAmPhenoAge clock^8^ and DNA methylation-based mortality risk assessment (DNAmGrimAge)^7^ were calculated using R scripts. Mean imputation was utilized for missing values. The first time point for each peripheral blood sample was used for epigenetic age estimation. For a subset of patients, technical replicates were present, and in these situations, we averaged the mean epigenetic age across replicates for all downstream analyses.

### Statistical Analyses

All statistical analyses were completed using R 4.1.2^23^. Longitudinal analyses of the effect of epigenetic age on disease progression were performed using Cox proportional hazard modeling with the R package ‘survival’^24^, controlling for *APOE ε*4 allele dosage, sex, years of education, and CDR-SB. Conversion to disease was analyzed in CN participants and was defined as a change in clinical diagnosis from CN to MCI or CN to AD. The proportional hazards assumption was tested for each model using the ‘cox.zph’ function and was not statistically significant (p > 0.05 level for all analyses shown). Mixed-effects linear regression analyses were used to assess the relationship between baseline epigenetic age and longitudinal MoCA scores, controlling for baseline and time interactions of baseline MoCA score, sex, years of education, and *APOE* ε4 dosage. We used the following linear mixed-effect model:

$$\text{ΔMoCA}=\beta_{0}+\beta_{1}\text{Δ}t+\beta_{2}{\text{MoCA}}_{\text{baseline}}*\text{Δ}t+\beta_{3}\text{Sex}*\text{Δ}t+\beta_{4}\text{Education}*\text{Δ}t+\beta_{5}APOE\varepsilon 4*\text{Δ}t+e$$

Associations between epigenetic age and cortical thickness were conducted using multiple regression after covarying for *APOE* ε4 dosage, sex, years of education, CDR-SB, and total intracranial volume. Volume-rendered images of multiple regression analysis results were created using the R package ‘fsbrain’^25^.

As has been done in prior work^26^ and in line with our hypotheses, we first performed all analyses using epigenetic age alone to predict disease progression and neuroimaging biomarkers. For outcomes which demonstrated a significant relationship at p < 0.05, we specifically tested whether epigenetic age provided independent information above and beyond chronologic age by covarying for chronologic age in addition to all previously listed covariates.

Where applicable, correction for multiple comparisons was completed using the false discovery rate (FDR) technique^27^.

### Approval and Consent

All ADNI research activities were approved by Institutional Review Boards at each patient recruitment site. Written informed consent was obtained from all patients.

## RESULTS

### Cohort

Data from 404 participants diagnosed as CN, MCI, or AD with DNA methylation data as well as quantitative neuroimaging data were included in this study (Table 1). The cohort was balanced with respect to sex and education (both p > 0.05) with expected differences in *APOE* ε4 dosage, CDR-SB score, and MoCA score (all p < 0.001). Interestingly, there was a significant difference (p = 0.02) in chronologic age, with the MCI group slightly younger (mean 73.2 years) than the CN (mean 75.3 years) and AD (mean 74.7) cohorts. As demonstrated in prior analyses of ADNI data, there were no significant differences between epigenetic age across groups for both DNAmPhenoAge (p = 0.69) or DNAmGrimAge (p = 0.13)^4^. A subset of study participants had processed FreeSurfer data available for analysis (n = 335, see Table S1 for additional details) and was overall similar in composition when compared to the parent cohort shown in Table 1.

### DNAmGrimAge and DNAmPhenoAge predict progression to MCI and AD

**Figure 1:**
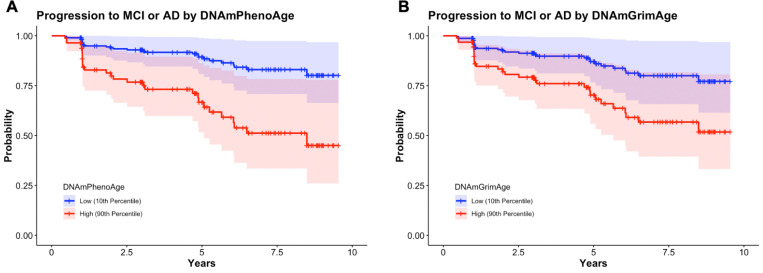
Survival plots stratified by epigenetic age (high = 90th percentile; low = 10th percentile) in participants who were cognitively normal at baseline are shown. DNAmPhenoAge significantly associated with progression to MCI or AD (**A**; p=0.006) and there was a trend towards significance DNAmGrimAge (**B**; p=0.06). Of note, DNAmPhenoAge significantly associated with progression to MCI or AD even after covarying for chronologic age (p=0.03). Shading represents 95% confidence intervals. Survival analyses were conducted using Cox proportional hazards modeling covarying for sex, education, CDR-SB score, and *APOE*ε4 dose; post hoc sensitivity analyses additionally covaried for chronologic age.

Analyses in this section utilized longitudinal clinical follow-up data from participants diagnosed as CN (n = 129) with progression defined as a change in clinical diagnosis from CN to MCI or CN to AD. Participants progressed to MCI or AD in 23.3% (n = 30) of the group over the study period. Using Cox proportional hazard modeling, we found that DNAmPhenoAge (Hazard Ratio [HR] 1.06; 95% confidence intervals [CI] 1.02–1.10; p = 3.45×10^− 3^) associated with conversion to MCI or AD (Fig. 1). There was also a trend towards significance for DNAmGrimAge (HR 1.03; 95% CI 1.00–1.06; p = 0.06) (Fig. 1). As a sensitivity analysis, we tested whether the effect of epigenetic age was independent of chronologic age and found that DNAmPhenoAge still significantly predicted progression in CN participants (HR 1.07; 95% CI 1.01–1.13; p = 0.01).

### DNAmPhenoAge predicts longitudinal cognitive changesduring normal aging

Given the findings above highlighting the importance of epigenetic age acceleration as a predictor of progression to MCI or AD, we next asked whether accelerated epigenetic age was also associated with longitudinal cognitive changes.

**Figure 2:**
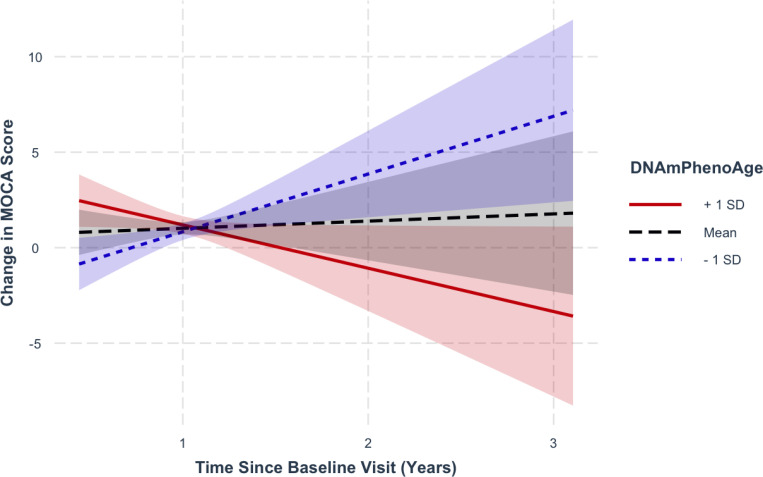
Regression plots in normal controls stratified by epigenetic age at representative levels (−1 standard deviation [SD] corresponding to the ~16th percentile; +1 SD corresponding to the ~84th percentile; and mean for reference). DNAmPhenoAge predicts decreased MoCA scores over time (p=4.26 × 10^−4^). This relationship remained significant after covarying for chronologic age (p=2.10 × 10^−3^). Shading indicates 95% confidence intervals. Linear mixed-effects analyses were performed covarying for sex, education, baseline MoCA score, and *APOE* ε4 dose.

Analyzing longitudinal MoCA scores in CN, we found that increasing epigenetic age as measured by DNAmPhenoAge associated with decreasing (i.e., worsening) MoCA scores over time (Fig. 2; β ± Standard Error (SE) = −0.30 ± 0.09; p = 4.26×10^− 4^). Importantly, this association persisted even after correcting for chronological age (β ± SE = −0.13 ± 0.04; p = 0.002). No associations were identified for DNAmGrimAge.

### Epigenetic age predicts neuroimaging markers of ADacross the spectrum of normal aging to AD

Given the associations between measures of increased epigenetic age and progression to MCI or AD, we next evaluated their association with neuroimaging biomarkers of AD, including whole-brain cortical thickness and WMH volumes. Due to differences in processing technique and data availability, only a subset of participants had cortical thickness measurements available (n = 335; see Table S1 for additional details). All participants in the survival analyses described above had WMH quantifications available for analysis.

**Figure 3:**
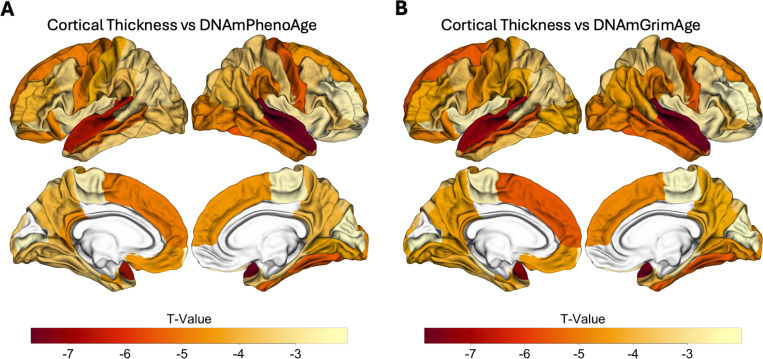
Epigenetic age predicts cortical atrophy across the spectrum of normal aging to AD. Both DNAmPhenoAge and DNAmGrimAge predicted cortical atrophy in AD-relevant regions such as entorhinal cortex, parahippocampal gyrus, and precuneus (all significant at an FDR corrected threshold of p_FDR_<0.05). Images were processed using FreeSurfer 5.1 and regions of interest were extracted from the Desikan-Killiany atlas. The relationship between cortical thickness and DNA methylation age was assessed using multiple regression covarying for sex, education, CDR-SB score, APOE ε4 dose, and total intracranial volume.

Across the spectrum of normal aging to AD, accelerated epigenetic age was associated with cortical thinning in nearly every brain region for 60 of 68 ROIs for DNAmPhenoAge and 59 of 68 cortical ROIs for DNAmGrimAge (p_FDR_<0.05; Tables S2 and S3; Fig. 3). Interestingly, the strongest associations were predominantly located in the temporal and parietal lobes in regions relevant to AD, such as entorhinal cortex and fusiform gyrus (Fig. 3).

To better understand the factors driving these findings, we conducted exploratory analyses examining DNAmPhenoAge and DNAmGrimAge within each diagnostic group and found these associations between accelerated epigenetic age and cortical atrophy were driven largely by the CN and MCI groups. For example, in the CN cohort there were associations for 42 and 47 of 68 cortical ROIs for DNAmPhenoAge and DNAmGrimAge, respectively (p_FDR_<0.05; Table S4). Similar findings were observed in the MCI group with associations for 50 and 47 of 68 cortical ROIs for DNAmPhenoAge and DNAmGrimAge, respectively (p_FDR_<0.05; Table S4). In contrast, the AD cohort showed no significant associations after correction for multiple testing.

We next repeated the above analyses while adding chronological age to the multiple regression model to better understand whether accelerated epigenetic age provided additional disease-relevant signal beyond that explained by advancing chronological age. In the CN subgroup, the association between epigenetic age and cortical thinning was mildly attenuated though remained significant at a p_FDR_<0.05 value for 14 of 68 ROIs for DNAmPhenoAge and 42 of 68 ROIs for DNAmGrimAge. Of note, many of the regions that remained significant after covarying for chronological age are those relevant to AD progression, including precuneus, cuneus, and fusiform gyrus.

Transitioning our analyses to examine WMH, we found that DNAmPhenoAge and DNAmGrimAge both strongly associated with WMH volumes in the combined cohort (p_raw_<0.001, Table 2) and when each diagnostic subgroup was considered individually (all p_raw_<0.05; Table 2). The associations between epigenetic age and WMH volumes were strongest in patients diagnosed with AD, where DNAmPhenoAge and DNAmGrimAge associated with WMH volume after covarying for chronologic age (p_raw_<0.05; Table 2).

## DISCUSSION

Among CN individuals, we found that accelerated epigenetic age associates with progression to either MCI or AD and with cognitive decline independent of chronological age. Furthermore, accelerated epigenetic age based on DNAmPhenoAge and DNAmGrimAge associated with cortical thinning in AD-relevant regions and WMH burden across the spectrum of normal aging to neurodegenerative disease. Interestingly, the relationship between epigenetic age and cortical thinning in AD-implicated regions appeared most robust in CN participants while the association between epigenetic age and WMH was greatest in participants with AD. Considered together, our findings suggest that advanced epigenetic age modulates risk for AD and cognitive decline even during their most nascent stages.

Our findings suggest that epigenetic age – as measured from peripheral blood – plays an important role in AD risk above and beyond that explained by chronological age, especially in CN individuals. This lends support to the hypothesis that the processes underlying biological aging contribute significantly to the development and progression of AD. Further, our data indicates that epigenetic age is more sensitive than chronological age in proxying this underlying biology. Indeed, even after covarying for chronologic age, elevated DNAmPhenoAge remained significantly associated with clinical progression, worsened MoCA scores, and cortical thinning. Given this, epigenetic age biomarkers may play a key role in identifying patients at risk for AD (and other diseases of aging) prior to symptom onset. For example, while amyloid can be detected up to 15 years prior to clinical symptoms^28^ it can be challenging to measure due to cost (e.g., PET scan^29^) or invasiveness (e.g., lumbar puncture^30^). n future clinical practice, estimates of epigenetic age may provide simple and minimally invasive metrics of AD risk that may also be relevant to other age-related conditions and diseases.

Building on prior work, our results suggest that employing a combination of analytic strategies, including longitudinal cognitive analyses and use of neuroimaging biomarkers of disease, may be useful for the validation of current and future epigenetic clocks. Of the prior studies showing a positive effect, one of the most conclusive also employed a survival analysis framework, though there was no significant association for DNAmPhenoAge^4^. While this study used cognitive and diagnostic data from the ADNI cohort, the authors did not perform survival analyses using ADNI data, nor did the study involve analysis of neuroimaging data. In addition, this study did not formally test whether epigenetic age was associated with longitudinal cognitive changes. Other promising studies examining epigenetics that have shown an association disease progression and neuroimaging biomarkers have focused on MCI rather than CN^31^ or have focused on overall mortality rather than AD^32^. Our findings suggest that analytic framework, rather than lack of a biologic relationship, may explain the seeming paucity of evidence linking findings from model organisms and human brain tissue to clinical populations.

We observed stronger associations between DNAmPhenoAge and disease progression whereas DNAmGrimAge associated most strongly with neuroimaging biomarkers. While speculative, we hypothesize that these differences are due to differential capture of AD-related risk factors by each epigenetic clock. For example, DNAmGrimAge differs from DNAmPhenoAge, a general lifespan clock, in that it was partially trained on serum proteins that predict mortality and smoking history^26^. As such, DNAmGrimAge strongly associated with time to death, time to coronary heart disease, time to cancer, and multiple other mortality-associated outcomes^26^ which may not be captured by DNAmPhenoAge but could be reflected by changes on a brain MRI. Evaluating the relative strengths and weaknesses of each generation of epigenetic clock and their subscores is beyond the scope of this article but may be a fruitful area for future research.

Our study benefits from our use of a thoroughly characterized cohort spanning the spectrum of normal aging to AD as well as our use of multiple strategies to test whether epigenetic age associates with AD risk. Limitations of our study include use of only two second-generation epigenetic clocks and lack of a suitable replication cohort given our multimodal approach. Our reliance on peripheral blood methylation data is both a strength, given its ease of acquisition, and a limitation, given it does not directly measure methylation in brain. However, data from multiple studies has shown that peripheral blood methylation changes are a reasonable proxy for brain with r > 0.85^33,34^.

In summary, we found that epigenetic age associates with progression to MCI or AD and cognitive decline in CN individuals as well as cortical thinning in AD-relevant regions and increased WMH across the spectrum of normal aging to neurodegenerative disease. Our analytic framework was critical to the successful identification of these associations and using a similar technique with other epigenetic clocks and neurodegenerative as well as other age-related diseases may be useful in future work. More generally, this study underscores the importance of advanced biologic age – above and beyond chronologic age – as a risk factor for AD and as a potential biomarker for clinical use.

## Supplementary Material

Supplement 1

## Figures and Tables

**Table 1:** Cohort Demographics

	CN	MCI	AD	
	121	236	47	p-value
Age at baseline (years; mean (SD))	75.3 (6.4)	72.8 (7.5)	74.7 (8.4)	0.01
Sex (Male (%))	57 (47.1%)	134 (56.8%)	30 (63.8%)	0.11
Education (years; mean (SD))	16.4 (2.8)	16.3 (2.7)	16.3 (2.5)	0.47
CDR-SB (mean (SD))	0.1 (0.3)	1.5 (1.1)	4.9 (2.2)	< 0.001
MoCA (mean (SD))	26.0 (2.4)	23.5 (3.3)	18.9 (4.6)	< 0.001
*APOE* ε4 dosage (Count (%))				
0	91 (75.2%)	131 (55.5%)	10 (21.3%)	
1	28 (23.1%)	82 (33.1%)	32 (63.8%)	<0.001
2	2 (1.7%)	23 (9.7%)	7 (14.9%)	

Summary statistics are shown for study participants summarized by diagnostic category with two-tailed p-values from ANOVA (continuous traits) or chi-square (categorical values) shown. CDR-SB, Clinical Dementia Rating scale Sum of Boxes; CN, cognitively normal; MoCA, Montreal Cognitive Assessment; MCI, mild cognitive impairment; AD, Alzheimer’s disease.

**Table 2:** Epigenetic Age Predicts White Matter Hyperintensity Volumes

	Primary Analysis			Covarying for Chronologic Age	
	Diagnosis (N)	Beta ± SE	p-value	Beta ± SE	p-value
DNAmPhenoAge	All Cohort (404)	0.32 ± 0.05	4.54E-09	0.13 ± 0.08	0.09
	CN (121)	0.39 ± 0.14	5.34E-03	0.27 ± 0.18	0.15
	MCI (236)	0.28 ± 0.06	5.13E-06	4.98E-3 ± 0.08	0.96
	AD (47)	0.41 ± 0.12	1.81E-03	0.44 ± 0.20	0.04
DNAmGrimAge	All Cohort (404)	0.49 ± 0.07	4.99E-10	0.18 ± 0.16	0.25
	CN (121)	0.51 ± 0.21	1.65E-02	0.17 ± 0.40	0.67
	MCI (236)	0.49 ± 0.08	1.79E-08	0.08 ± 0.17	0.63
	AD (47)	0.54 ± 0.17	2.94E-03	0.98 ± 0.43	0.03

White matter hyperintensity (WMH) volume associates with two key measures of epigenetic age (DNAmPhenoAge and DNAmGrimAge) across the spectrum of normal aging to neurodegenerative disease. In our primary analysis, epigenetic age associated with WMH volume in the overall cohort as well as within each diagnostic subgrouping available for analysis. In our sensitivity analyses covarying for chronologic age, epigenetic age remained a significant predictor of WMH volume in Alzheimer’s disease. All analyses were conducted using multiple regression covarying for *APOE* ε4 status, sex, years of education, CDR-SB, and total intracranial volume. AD – Alzheimer’s disease, CN – cognitively normal, MCI – Mild cognitive impairment, SE – Standard Error.

## Data Availability

Data from ADNI is available after application through a publicly accessible research portal (ADNI: adni.loni.usc.edu/data-samples/access-data/).
