# Supplementary material for: DNA methylation age from peripheral blood predicts progression to Alzheimer’s disease, white matter disease burden, and cortical atrophy
Source: Res Sq. 2024 Oct 28:rs.3.rs-5273529. Preprint. [Version 1] doi: 10.21203/rs.3.rs-5273529/v1 (PMC11581046; doi:10.21203/rs.3.rs-5273529/v1)
Supplement: Supplement 1 [file NIHPPRS5273529V1-supplement-1.pdf]

Supplementary Files

This is a list of supplementary files associated with this preprint. Click to download.

- SupplementaryTables.docx
